# Cluster analysis of aging and sexual well-being: Insights from Portuguese and Spanish older adults

**DOI:** 10.1192/j.eurpsy.2025.885

**Published:** 2025-08-26

**Authors:** S. von Humboldt, E. Cabras, G. Low, I. Leal

**Affiliations:** 1 William James Center for Research, ISPA – Instituto Universitário, Lisbon, Portugal; 2 Department of Education, Universidad Alfonso X el Sabio, Madrid, Spain; 3 Faculty of Nursing, University of Alberta, Edmonton, AB, Canada

## Abstract

**Introduction:**

Aging well in a cross-cultural perspective may encompass pertinent challenges in terms of adjustment, sexual well-being and satisfaction with life in the late years.

**Objectives:**

Considering the paucity of empirical data concerning cultural diversity of experiencing aging, this study aims to help fill this gap by assessing the specific patterns of sexual satisfaction, adjustment to aging (AtA), and life satisfaction with life (SwL) of older adults in Portugal and Spain.

**Methods:**

This cross-national study included 443 older adults, aged 65 and older, from Portugal and Spain. Five instruments were applied: (a) Adjustment to Aging Scale (ATAS); (b) Satisfaction with Life Scale (SwLS); (c) New Sexual Satisfaction Scale - Short (NSSS-S); (d) Mini-Mental State Exam; and (e) Sociodemographic, health and lifestyle questionnaire. K-means cluster analysis was employed to identify and characterize the clusters considering adjustment to aging, sexual satisfaction and life satisfaction. One-way ANOVAs were conducted to analyze differences for sexual-well-being among clusters.

**Results:**

Findings indicated three clusters, which explained 79.3% (*R-sq* = 0.793) of the total variance: Cluster #1: “Well Adapted” (*n* = 37, 8.8%), Cluster #2: “Struggling to Adapt” (*n* = 141, 31.8%), and Cluster #3: “Active Agers” (*n* = 265, 59.8%). Participants in Cluster #1 were mostly Portuguese, with high levels of AtA, sexual satisfaction, and SWL. Conversely, Cluster #2 integrated mostly Portuguese participants with moderate sexual satisfaction, and lower levels of AtA and SwL. Participants from Cluster #3 were mostly Spanish with moderate levels of AtA and reduced sexual satisfaction and SwL.

**Conclusions:**

This study innovates by exploring the elaborate interplay among sexual satisfaction, AtA, and SwL in a cross-cultural perspective, with implications for tailoring interventions, service planning, development and evaluation of culturally-diverse older populations.

**Keywords:**

Adjustment to aging; cluster analysis; older adults; satisfaction with life; sexual satisfaction.

**Disclosure of Interest:**

None Declared

